# The neural substrates of bruxism: current knowledge and clinical implications

**DOI:** 10.3389/fneur.2024.1451183

**Published:** 2024-10-01

**Authors:** Karin Harumi Uchima Koecklin, Aron Aliaga-Del Castillo, Peng Li

**Affiliations:** ^1^Life Sciences Institute, University of Michigan, Ann Arbor, MI, United States; ^2^Department of Biologic and Materials Sciences, School of Dentistry, University of Michigan, Ann Arbor, MI, United States; ^3^Department of Orthodontics and Pediatric Dentistry, School of Dentistry, University of Michigan, Ann Arbor, MI, United States; ^4^Department of Molecular and Integrative Physiology, University of Michigan Medical School, Ann Arbor, MI, United States

**Keywords:** brain, bruxism, neural substrates, trigeminal, jaw muscles, orofacial behavior, neuroimaging

## Abstract

Bruxism is a complex orofacial behavior that can occur during sleep or wakefulness, characterized by the involuntary grinding or clenching of teeth, involving repetitive activity of the jaw muscles. Its etiology is multifactorial, influenced by genetic, psychological, physiological, and lifestyle factors. While the mild bruxism may not necessitate treatment, severe bruxism can lead to significant consequences, including tooth damage, jaw pain, fatigue, and headaches. The bruxism has been associated with medical conditions, such as stress, anxiety, sleep disorders, and various neurological disorders; however, the exact pathophysiology remains elusive. Although the central nervous system is strongly implicated in the development of bruxism, specific neural substrates have not yet been conclusively established. Furthermore, there is evidence to suggest that individuals with bruxism may exhibit neural plasticity, resulting in the establishment of distinct neural circuitry that control the jaw movements. The application of various neurophysiological techniques in both clinical and pre-clinical studies provides valuable insights into the neural mechanisms underlying bruxism. This review aims to comprehensively examine the current literature on the neural pathways involved in bruxism, with the goal of improving the clinical approach and therapeutics for this condition. A deeper understanding of the neural circuitry controlling bruxism holds the potential to advance future treatment approaches and improve the management of patients with bruxism.

## 1 Introduction

Bruxism is defined as repetitive masticatory muscle activity characterized by clenching or grinding of the teeth, and/or by bracing or thrusting of the mandible, according to an international consensus in 2013 (1). Bruxism is a parafunctional activity that is commonly observed, with a prevalence estimated to affect up to 30% of the population (2, 3). This condition can occur either during the day or at night, known as awake bruxism and sleep bruxism, respectively (4–6). While sleep bruxism is defined as a masticatory muscle activity during sleep, characterized as rhythmic (phasic), non-rhythmic (tonic), and is not a movement disorder or a sleep disorder in otherwise healthy individuals; awake bruxism is defined as a masticatory muscle activity during wakefulness, characterized by the repetitive or sustained tooth clenching and grinding, as well as mandibular bracing and thrusting, and is not a movement disorder in otherwise healthy individuals (5, 7). Current consensus considers bruxism a behavior that is a risk factor for many health conditions, including not only tooth wear and prosthodontic complications, but also serious masticatory muscle pain and temporomandibular joint (TMJ) pain (5, 8–10).

Despite regular dental examinations aiding early detection and management of bruxism, current treatments are limited to reducing symptoms and preventing damage without targeting the underlying cause. For instance, occlusal splints or mouthguards are used to intervene symptomatically on sleep bruxism, but they may lead to an increase in clenching or grinding activity (11, 12). Medications, such as botulinum injections, can be prescribed to alleviate muscle activity and pain. However, these injections only decrease the strength of masticatory muscle contractions but do not decrease the incidence of sleep bruxism events (13). Therefore, it is essential to understand the mechanisms of bruxism to develop better treatments targeting the causes of this condition.

Bruxism presents a multifactorial etiology (14). The development of bruxism likely involves genetic, psychological, physiological, and lifestyle factors. Genetic predisposition may play a role, as bruxism can run in families, suggesting a hereditary component (15). Psychological conditions, such as stress and anxiety, have been highly related to bruxism (16). Physiological factors, including sleep disorders, abnormal neurotransmitter levels, and certain medications, can also contribute to the development of bruxism (17, 18). Lifestyle factors like the consumption of alcohol, caffeine, tobacco, and drug abuse have also been associated with bruxism (19). All of these factors converge on the excessive masticatory muscle activities, so it would be valuable to identify the neural pathways involved in bruxism (20). Although the prevalence of bruxism in children and adolescence is reported to be as high as 40–50%, these estimates often rely on parental reports and encompass a wide age range of participants (3, 21, 22). Furthermore, most studies on the neural circuitries involved in bruxism have been conducted in adults. Given that the adult brain’s circuitry is fully mature and developed, focusing on adults allows for a clearer understanding of the neural pathways involved in this complex behavior.

The use of different modern tools for the evaluation of the neural pathways involved in bruxism in both clinical and animal studies can provide a deeper understanding in this topic. The aim of this review is to comprehensively examine the current literature on the neural pathways involved in bruxism. The uncovering of the neural control involved in this behavior can provide important information for the development of future therapeutic approaches and prevent the consequences of severe bruxism.

## 2 Methods

The following databases were searched for article selection: PubMed, Scopus, Science direct, and EBSCO host until December 2023. The search strategies are detailed in Table 1. The search strategy was modified for Science direct and EBSCO host to improve the number of articles obtained. Additionally, filters were applied to Science direct to narrow the search and avoid the search of tertiary sources and gray literature. To secure a deep search and retrieve more relevant articles related to the topic, a manual search was performed in Google scholar and the bibliographic references of the included studies. There were no restrictions on language or date of publication. Both human and animal studies related to the neural control of bruxism, muscle hyperactivity, tooth grinding or clenching were considered for this review, while duplicate studies and review papers were excluded. A total of 4,751 articles were found, with 837 duplicate articles excluded. Article selection by title and abstract included 179 articles. Finally, after reading the full-text articles, a total of 23 animal studies and 72 clinical studies were included in this review.

**Table 1:** Search strategy.

Article search	Search strategy	Filter	Number of articles
PubMed	(bruxism OR grinding OR clenching OR Brux) AND (neural plasticity OR functional MRI OR neural control OR neural changes OR brain OR neuroscience)	None	1,203
Scopus	(bruxism OR grinding OR clenching OR brux) AND (“neural plasticity” OR “functional mri” OR “neural control” OR “neural changes” OR brain OR neuroscience)	None	826
Science direct	(bruxism OR grinding OR clenching) AND (“neural plasticity” OR “neural control” OR neuroscience)	Review Research article	2,170
EBSCO host	(bruxism OR grinding OR clenching) AND (“neural plasticity” OR “neural control” OR neuroscience)	None	525
Manual selection			27

## 3 Brain regions involved in jaw movement and bruxism

### 3.1 Motor neurons

The masticatory muscles, vital for tasks like chewing and clenching, are governed by motor neurons in the trigeminal motor nucleus (Vmotor) within the pontine region of the brainstem. These muscles exhibit distinct patterns of activity depending on the function they perform, such as during normal chewing, forceful clenching, or bruxism (23). Studies have shown that the excitability of the temporalis muscle, a key masticatory muscle, can be assessed through H-reflex measurements. Svensson et al. demonstrated that H-reflex amplitudes increase with the intensity of clenching tasks, indicating heightened neural excitability during stronger muscle contractions (24). Clenching movements require isometric muscle contraction, while closing movements require isotonic muscle contraction. These different muscle contractions may produce more muscle effort during clenching, compared to jaw opening and closing tasks. The differences in motor control strategies during clenching could potentially lead to decreased inhibitory jaw reflexes specific to this task (23).

Motor neuron activity during voluntary clenching in patients with bruxism may differ from normal conditions. During voluntary contractions, trigeminal motoneurons activate Persistent Inward Currents (PICs). The onion skin effect, where higher threshold units fire at lower rates than lower threshold units, along with PIC activation, facilitates sustained activation of the masseter muscle. Evaluation of these parameters have shown that the motor neuronal activation during voluntary clenching in bruxism patients seemed to be similar to non-brux patients (25). However, nerve growth factor-induced sensitization associated with motor training task have been reported to have no significant effect in the central modulation of motor pathways in bruxers, as opposed to non-bruxers (26).

Sleep-bruxism patients present both lower levels of the masseter motor-evoked potentials and the masseter inhibitory reflex, with possible altered interneuronal activity necessary for the processing of non-nociceptive information (27, 28). This suggests that an abnormal motor control of the masticatory muscles during bruxism, due to disrupted interneuronal activity, could lead to a reduced excitability of the inhibitory circuitry in the jaw motor system.

### 3.2 Cerebellum

The cerebellum, a crucial brain structure for motor coordination and cognitive processing, has been shown to activate during clenching with natural teeth in functional MRI studies (29–31). Similarly, voluntary clenching induced by a visual stimulus has demonstrated either bilateral or unilateral cerebellar activation prior to clenching, suggesting the involvement of the cerebellum in the cognition process of orofacial motor tasks (32). These findings suggest that the cerebellum plays a role not only in motor control but also in the higher-order cognitive processing required for complex motor behaviors like clenching.

In a study on mice utilizing Ca^2+^ imaging to identify the neuronal activity, researchers observed that the stellate cells (inhibitory interneurons of the cerebellum molecular layer) were active during bruxing behaviors in freely moving animals (33). These stellate cells were specifically activated in the Crus II area of the posterior lobe of the cerebellum. This area was also active during licking, suggesting a link between the Crus II area and orofacial muscle activity. However, while the stellate neurons activated during licking presented an organized spatial distribution along the parallel fiber axis, those activated during bruxing lacked spatial organization. Interestingly, the cerebellar basket cell interneurons activated during bruxing presented high levels of coordination, and their Ca^2+^ signal strength was similar to the that of the stellate neurons.

### 3.3 Higher brain areas

#### 3.3.1 Higher brain areas in clenching and brux behaviors in human

The higher brain areas involve the parts of the brain that perform higher-level functions necessary for complex behaviors, including learning, memory, sensory integration, and decision-making activities (34). Involvement of higher brain areas, including the cerebral cortex, during clenching and bruxism behaviors underscores the complex neural processes associated with these parafunctional masticatory tasks (35). Functional MRI studies have revealed robust activation patterns across various cortical regions in response to different types of clenching stimuli, highlighting the intricate interplay between sensory, motor, and cognitive pathways (35–37).

The inferior frontal gyrus, including the prefrontal area and Brodmann’s area (BA) 44 and 45, is activated during clenching with natural teeth (30). Interestingly, the visual cortex (BA 17, 18) is also active during clenching with soft splints. In contrast, clenching with a hard splint activates additional areas such as the supplementary motor area (SMA, BA 6) and the temporal association area (TAA, BA20, 37), leading to higher masseter activity compared to clenching with natural teeth (30). Increased clenching force in healthy subjects activates these areas and additional regions like the primary sensorimotor cortex (SMC, BA 1, 4), and the temporal association area (BA 20–22, 37) (29). These results suggest that the proprioceptive information from orofacial tissues may be involved in these differences, affecting the striate and parastriate areas.

Other cortical areas activated during teeth clenching include the pre-central gyrus, post-central gyrus, sensory cortex, motor cortex, pre-motor cortex, somatosensory cortex, prefrontal cortex, temporal cortex, frontal operculum, basal ganglia (putamen), parietal cortex, cingulate cortex, and insula (31, 38–40).

Although the motor and premotor cortex present high activation during clenching tasks, the activation pattern depends on the type of clenching task. Intercuspal clenching tasks show higher area and volume activation compared to incisor tooth clenching task (41). This suggests that the differences in proprioceptive information from the periodontal ligament of the anterior and posterior teeth could be involved. It is possible that the differences between the centric and eccentric movements in parafunctional jaw movements and bruxism correspond to different activation patterns in the brain.

Brain activity differences between left and right hemispheres during clenching tasks have been found in different subjects, with asymmetrical activations in the motor, premotor, and somatosensory cortices (37). Chewing-side preferences may be reflected in hemispheric dominance, and neural plasticity due to long term habits could involve both the primary sensorimotor cortex and primary motor cortex (42).

Pathological brain activity has also been associated with abnormal jaw activity. Tooth clenching has been linked to triggering migraine (43). Acquired brain injuries, such as traumatic brain injury, and sub arachnoid hemorrhage, are associated with increased parafunctional activity of the masticatory muscles (44). Neurological conditions like epilepsy have shown connections with bruxism as well (45), with bruxism episodes reported during temporal lobe seizures (46). However, the exact relationship between these pathological neural conditions and bruxism remains unclear.

#### 3.3.2 Cortical involvement in the generation of brux-like behaviors in animals

Cortical stimulation can induce bruxism-like behaviors in animals (47, 48). In a study with rabbit, stimulation of the brain cortex surface around the chewing area under anesthesia elicited bruxing behaviors (47). Bruxing was induced by high-frequency electrical stimulations in the anteromedial region of the cortex, specifically the jaw motor cortical area. The regular rhythm of the bruxing behaviors were 3–4 cycles per second, regardless of the stimulation frequency, suggesting that abnormal excitation of the jaw-motor cortical area plays a role in the development of bruxing behaviors. Other animal studies have showed that structural disease conditions in the forebrain can lead to awake bruxism in dogs (49).

Higher processing areas in the brain, such as the central nucleus of the amygdala (CeA), are connected to Vmotor in humans. The CeA is involved in the processing of stress, and it has been suggested that the CeA–Vmotor circuit may be related to aberrant jaw motor function in humans (50). Interestingly, the CeA also increases its activity while when performing masticatory behaviors to cope with stress in rodents (51).

#### 3.3.3 Differences in neural activation of patients with bruxism

Repeated clenching tasks can trigger neural plasticity in the corticomotor circuitry involved in jaw-closing muscle activation, as evidenced by motor-evoked potential facilitation of the masseter muscle and increased cortical excitability (52). Similarly, sleep bruxism is associated with neural plasticity changes, affecting motor force control and masticatory motor learning (53). The installation of the aberrant orofacial movements may provide the basis for the neural adaptability changes in patients with bruxism.

Decreased cortical activation patterns have been reported in patients with bruxism during voluntary clenching tasks, particularly showing less activation in the right inferior parietal lobule and dorsal posterior cingulate area (54). This suggests that patients with bruxism may have less controlled movements of the masticatory muscles.

In addition to the cortical activation, subcortical activation pattern is also different in patients with parafunctional brux behaviors. Patients with parafunctional tooth grinding and clenching exhibit a narrower activation pattern in the SMA, pre-SMA (the most anterior section of SMA), and left inferior parietal lobule during voluntary clenching and grinding compared to normal subjects (55). Similarly, SMA, pre-SMA, proSMA (posterior part of SMA, or proper SMA), and SMC areas present lower activation during clenching tasks in subject with brux behaviors compared to normal subjects. During grinding tasks, SMA, pro-SMA, the Rolandic operculum, and the putamen, show lower activation in patients with brux behaviors (35). It is implied that patients with these pathological jaw movements have less extensive activity patterns due to the habitual execution of these over-learned tasks, resulting in more efficient and controlled jaw muscle movement (55).

Patients with sleep bruxism exhibit changes in the neuronal excitability in the corticomotor control of the jaw-closing muscles. Motor-evoked potentials of the masseter and cortical area activation are lower in patients with sleep bruxism, while motor force control and masticatory motor learning are also affected (53). Conversely, increased cortical activation in the primary sensory motor cortex (BA 4), has been reported during clenching and chewing in patients with confirmed sleep bruxism, compared to healthy patients. However, no changes in the masticatory muscle activity were found (56). The authors suggested that patients with sleep bruxism may not have special trained skills for these tasks, thus showing an increased cortical activation. Another explanation proposed was that enhanced parafunctional activity with more recruitment of the tongue muscles, accompanied by jaw movements, could correspond to the increase in cortical activation. The authors also recommended confirmatory diagnosis of sleep bruxism with polysomnography rather than self-reported or oral evaluation of bruxism.

Patients with sleep bruxism also present abnormal excitability in the neural masticatory pathways compared to control subject, with possible involvement of neurotransmitters such as serotonin. These changes in neuronal excitability may be influenced by brainstem area, rather than the cortex (57).

## 4 Somatosensations and bruxism

Bruxism involves complex interactions between sensory and motor pathways, including proprioception and nociception. Understanding these mechanisms can provide insights into the pathophysiology of bruxism and potential therapeutic targets.

### 4.1 Proprioception in bruxism

Jaw tremor at a frequency of 6–10 Hz has been identified as potential biomarker of bruxism in humans. This increased jaw tremor in patients with sleep bruxism may be attributed to increased afferent feedback from the mechanoreceptors in the periodontal ligament (58).

Proprioception information from the periodontal ligament has been linked to the development of bruxism-like behavior in animal models. Mechanoreceptors in the periodontal ligament transmit the sensory information during grinding movements, allowing molar teeth to sense the magnitude and direction of the forces generated during tooth grinding (48).

The mesencephalic nucleus of the trigeminal nerve (Vmes) is crucial for processing the proprioceptive information from muscle spindles and the periodontal ligament (59). Vmes is a premotor area of Vmotor and contains excitatory neurons expressing the vesicular glutamate transporter 1 (VGLUT1). It has been suggested that Vmes is involved in the generation of masseter hypercontraction in mice. Studies using malocclusion models, such as anterior crossbite in rats, have shown high levels of VGLUT1 expression in Vmes, the periodontal ligament, Vmotor, and the masseter muscle. Increased acetylcholinesterase, a marker for muscle activity, was also observed in the masseter (60). These findings suggest that Vmes-mediated VGLUT1 expression plays a role in masseter hypercontraction.

A model of chronic restraint stress in mice showed increasing anxiety-like behaviors accompanied by masseter hyperactivity and enhanced excitability of Vmes neurons. Downregulation of VGLUT1 in Vmes reduced acetylcholinesterase and creatine kinase muscle-type levels in the masseter muscle, indicating reduced masseter hyperactivity (61).

Another malocclusion study showed development of anxiety-like behaviors and increased activity levels in the lateral habenula (62), a brain area involved in the processing of stress, anxiety, depression, and aversive stimuli (63, 64). Most efferent neurons in the lateral habenula are glutamatergic, expressing the vesicular glutamate transporter 2 (VGLUT2) (65). Furthermore, the lateral habenula neurons sent projections to Vmes, and increased VGLUT2 synaptic vesicles were found around the caudal portion of Vmes neurons after inducing a unilateral anterior crossbite. Downregulation of VGLUT2 expression in the lateral habenula also decreased Vmes activity (62).

Vmes neurons also receive projections from neurotransmitter systems implicated in bruxism. Caudal and to a lesser extent, rostral Vmes neurons receive dopaminergic, noradrenergic, GABAergic, and serotonergic fibers (66). Additionally, Vmes premotor neurons of Vmotor receive dense tyrosine hydroxylase innervation, possibly from the locus coeruleus, suggesting its potential role in generating bruxism-like behaviors (67).

### 4.2 Nociception in bruxism

Nociceptive processing in masticatory muscles may differ between bruxers and non-bruxers. Patients with bruxism may not show changes in masseter corticomotor excitability after noxious stimuli combined with masticatory-muscle training tasks, unlike control subjects who exhibit changes in corticomotor excitability (26).

In a study in anesthetized rats, electrical stimulation of the masseter muscle induced teeth clenching, suggesting a role for sensory innervation from the muscle in teeth clenching. Moreover, administration of dantrolene, a muscle relaxant, decreased the force of the teeth clenching, indicating the involvement of sensory information from orofacial structures in the neuropathogenesis of brux behaviors (68).

## 5 Neurotransmitters involved in bruxism

### 5.1 Glutamate and gamma-aminobutyric acid (GABA)

Both glutamatergic (excitatory) and GABAergic (inhibitory) systems could be affected in patients with bruxism. A study on occlusal splint users with possible bruxism showed that lower levels of GABA is and higher levels on Glutamine metabolites are present in the dorsolateral prefrontal cortex, while only GABA is increased in the thalamus, compared to the control group (69). On the other hand, decreased levels of GABA were found in the brainstem of subject with possible diagnosis of sleep bruxism (70), suggesting that the inhibitory control of the different nuclei in brainstem could be affected by this condition.

Disturbances in glutamatergic and GABAergic systems have been related to sleep bruxism. A negative feedback circuit between hippocampus and dorsolateral prefrontal cortex (DLPFC) might play an important role in regulating bruxism behaviors (69). Lower levels of GABA+ have been reported in the brainstems of potential sleep bruxism patients, suggesting that sleep bruxism might be primarily influenced by brainstem networks rather than cortical networks. Additionally, brainstem GABAergic networks might be a potential treatment target for this condition (70). However, well conducted clinical trials are necessary to validate these findings.

### 5.2 Serotonin

Serotonin is another neurotransmitter associated with bruxism, known for its role in regulating muscle tone during sleep and maintaining arousal states (71). Genetic studies have linked polymorphisms in serotonergic neurotransmission to an increased risk of sleep bruxism, such as the C allele of the HTR2A rs6313 single nucleotide polymorphism (SNP), which is associated with a 4.25 times higher risk of sleep bruxism, due to reduced expression of 5-HT2A receptors (71). *In vitro* studies using human induced pluripotent stem cells-derived neurons carrying the HTR2A C allele further revealed altered membrane properties, including increased action potential frequency and decreased half-duration, indicating heightened neuronal excitability in sleep bruxism patients (72).

Selective serotonin reuptake inhibitors (SSRIs), commonly used for chronic depression, have also been linked to the development of sleep bruxism. SSRIs like paroxetine, citalopram, and fluvoxamine have been shown to associate with increased incidence of bruxism (73–78). Furthermore, experimental studies in mice with citalopram injections demonstrated alterations in masseter muscle activity during non-REM sleep, indicating a potential role of serotonin in motor control during this phase, though not affecting the overall sleep–wake cycle distribution (79).

### 5.3 Dopamine

The dopaminergic system has also been suggested to be involved in the pathophysiology of bruxism. Dopamine plays a crucial role in motor control, and its dysregulation is observed in pathological conditions affecting motor functions, such as Parkinson’s disease (80, 81).

In sleep bruxism, an imbalance in striatal dopamine receptor-2 (D2R) expression has been observed, with a unilateral decrease in D2R levels (82). Treatment with the D2R agonist bromocriptine has been shown to reduce the number of sleep bruxism episodes and correct the imbalance in D2R distribution (83). For patients with sleep/awake bruxism, reduced frontal lobe activity has been noted. These patients respond to metoclopramide, a selective D2R antagonist, which decreases bruxism episodes. This suggests hypersensitivity to presynaptic D2R in the prefrontal cortex, indicating a distinct etiopathogenesis from sleep bruxism (84).

Genetic studies have also linked single nucleotide polymorphisms (SNPs) in dopaminergic pathway genes to bruxism. The G allele of the DRD2 rs1800497 polymorphism is associated with a decreased risk of awake-sleep bruxism, possibly due to enhanced dopaminergic function. Similarly, the C allele of the DRD5 rs6283 polymorphism is linked to a decreased risk of awake bruxism, potentially due to changes in D5 receptors. Conversely, the C allele of the DRD3 rs6280 polymorphism is associated with an increased risk of sleep bruxism, likely due to increased dopamine release (6). These findings suggest that variations in dopamine levels may influence whether bruxism occurs during sleep, wakefulness, or both.

Parkinson’s disease (PD) is characterized by the degeneration of dopaminergic neurons in the substantia nigra pars compacta, leading to symptoms such as muscle rigidity and tremor (81). Patients with PD often experience increased episodes of bruxism during both sleep and wakefulness, suggesting a potential link between dopaminergic dysfunction and bruxism (85, 86).

The association between dopamine and bruxism has also been demonstrated in animal studies. In a study with rats, injection of the dopamine receptor agonist combination of SKF 38393 and quinpirole into the ventral striatum induced periodic episodes of audible teeth grinding at the later phases of the drug action. This grinding was accompanied by continuous activity in both the masseter and anterior digastric muscles during the peak drug effect. Interestingly, the rats also exhibited varied oral movements, including jaw opening and closing, lateral movements, and frequent tongue protrusion. In contrast, injection of the acetylcholine receptor agonist carbachol did not produce any teeth grinding (87).

Although the association between bruxism and malocclusion in humans is not strong (88), occlusal disharmonies and malocclusion have been associated with bruxism behaviors in animal models. Acute occlusal disharmonies in the lower incisors of rats have shown increase of DOPA levels in the striatum, frontal cortex, and hypothalamus, with dopamine increase in the hypothalamus, and both dopamine and noradrenaline in the frontal cortex (89).

Stress-induced non-functional masticatory activity in rats resulted in higher levels of extracellular dopamine in the prefrontal cortex, correlating with the severity of the activity. Additionally, stress-induced non-functional masticatory activity led to increased 3,4-Dihydroxyphenylalanine (DOPA) accumulation and decreased dopamine breakdown in the striatum (90, 91).

## 6 Other behaviors associated to bruxism

### 6.1 Stress and anxiety

Bruxism is a multifactorial condition whose etiology remains unclear, involving various psychological and physiological factors (92). Psychological traits, such as depressive symptoms, manic tendencies, stress, and anxiety have been closely associated with bruxism (93). Patients with mood disorders exhibit significantly higher odds of developing bruxism, underscoring the role of mental health in its pathogenesis (94).

Stress, in particular, has been implicated in disrupting neural synapses and connectivity, potentially exacerbating bruxism symptoms (95). Specific anxiety symptoms such as panic, stress sensitivity, and coping capability deficits are also linked with bruxism (96, 97). Opipramol, a tricyclic antidepressant, has been shown to reduce episodes of sleep bruxism, highlighting the therapeutic relevance of managing underlying anxiety (98).

Experimental studies in rodents have demonstrated that increased masticatory muscle hyperactivity resembling brux behaviors. Tooth clenching has been observed to impact cardiac output and activate the sympathetic nervous system, potentially linking it to stress responses (99). Interestingly, administration of diazepam, an anxiolytic drug that enhances GABA-receptors, reduces bruxism-like behaviors in rats, suggesting a modulation of masticatory muscles hyperactivity by emotional state (100). Higher brain areas implicated in stress regulation, such as the amygdala or thalamus, along with changes in the GABAergic system, may be involved in the development of bruxism under stress conditions.

Chronic malocclusion, such as unilateral anterior crossbite, has been shown to increase anxiety-like behaviors in rodents. Excitatory neurons expressing VGLUT1 in the dorsomedial part of the principal sensory trigeminal nucleus (Vpdm), a key area in the trigeminal system and postsynaptic to Vmes, appear pivotal in the anxiety induced by malocclusion. Inhibition of these VGLUT1+ neurons in Vpdm alleviates malocclusion-induced anxiety, while their activation alone induces anxiety-like behaviors irrespective of malocclusion presence (101). Moreover, Vmes neurons transmit proprioceptive information to Vdpm, whereas Vpdm neurons project to the ventral posteromedial nucleus (VPM). Activation of VPM neurons, which receive innervation from Vpdm, correlates with anxiety generation, suggesting a neural pathway where VPM activation may contribute to anxiety-like behaviors induced by malocclusion. Given that both anxiety and malocclusion models in rodents demonstrate masseter muscle hyperactivity, proprioception processed in Vpdm could play a role in the development of bruxism-like behaviors.

Restraint stress models in rodents also induce bruxism behaviors, with behaviors like wood chewing and gnawing observed to alleviate these conditions (61, 102–104). The paraventricular hypothalamic nucleus (PVH) is a brain area involved in the regulation of trigeminal system neuronal activity during stress, with chronic restriction stress models showing PVH activation. PVH also shows projections to the trigeminal system brain areas, like Vmotor, Vmes and the peritrigeminal premotor area (Peri5). Studies suggest that PVH-Peri5 neural connections may contribute to bruxism-like behaviors following restraint stress in mice, with inhibition of PVH neurons projecting to Peri5 reducing gnawing behaviors (101).

Other brain areas associated with stress and emotional control, like CeA, lateral hypothalamic area, and parasubthalamic nucleus, directly or indirectly activate Vmotor by projecting to Peri5, Vmes, and the supratrigeminal nucleus (105).

### 6.2 Sleep–wake states and bruxism

#### 6.2.1 Sleep–wake regulation of masticatory muscle activity

The sleep wake states play a role in the activity of the masticatory muscles. In a study in guinea pigs, the overall masseter and neck muscles activation were recorded during sleep–wake cycles. While the neck muscles activity was decreased during non-REM, and almost not present during REM sleep, the magnitude of the masseter activity during both REM and non-REM sleep was five times lower than during wakefulness. Overall, the activation patterns of the masseter muscles during sleep–wake cycles differ from those observed in the neck muscles, indicating that motor activation and inhibition in the masticatory muscles may be regulated distinctly during sleep (106).

This distinct regulation was also observed in mice, where the activity of the masseter and neck muscles activity showed correlation during non-REM sleep, but this correlation was absent during REM sleep. During REM sleep, phasic twitches are frequently found for the masseter muscle but not the neck muscles. Moreover, the masseter activity during non-REM sleep was bimodal, with higher activity during arousal, while the neck muscles only present unimodal firing activity. Similar patterns were observed during wakefulness, where bimodal activation of the masseter muscle reflected its dual role in fine jaw movement control with low-level contraction, as well as high-level contraction for tasks requiring more muscle force (107).

#### 6.2.2 Bruxism and sleep architecture

Sleep bruxism episodes and tooth grinding during sleep have been proposed as manifestations of micro-arousal or awakening, due to a response to cortical arousal (108, 109). Rhythmic masticatory muscles activities (RMMA) episodes observed in patients with sleep bruxism are preceded by cortical EEG changes, followed by heart rate acceleration. These RMMA events have also been associated with concurrent movements of the legs and neck, indicating that these orofacial muscle activations may occur in response to micro-arousals (110). The study proposes that RMMA are preceded by increased brain activity and autonomic nervous system activation. Given the involvement of heart rate changes and the autonomic system activity in sleep bruxism, the use of clonidine, an antihypertensive drug, has been shown to reduce the episodes of sleep bruxism, possibly due to suppression of the sympathetic innervation (111).

Patients with severe sleep bruxism present a shortened rapid-eye movement (REM) sleep latency and percentage of occurrence, and an increase in the number of the sleep stage transitions (112). REM sleep and sleep stage N2 seem to be fragmented in patients with sleep bruxism however, this was not related to RMMA episodes. RMMA may be present during long runs of N3 sleep stage, suggesting that this muscle activation is related to dissipation of the sleep pressure (113). Furthermore, while sleep bruxism may be associated with insomnia, RMMA episodes do not seem to be the cause (114). These results may imply that patients with sleep bruxism present altered sleep architecture, possibly related to REM sleep, regardless of RMMA episode occurrence.

Sleep bruxism episodes are observed to be more frequent during the non-REM sleep stage 1 or 2 compared to REM sleep, suggesting a stage-specific occurrence pattern (109, 115). Patients with bruxism also exhibit altered theta wavelength activity during sleep (116). The microstructural aspect of sleep are affected in individuals with sleep bruxism, characterized by reduced K-complexes and K-alpha index during the non-REM stage 2 (115).

Interestingly, patients with severe sleep bruxism accompanied by temporomandibular joint and masticatory muscle pain, are more likely to present brux episodes during REM sleep, compared to patients without severe bruxism (117). Similarly, there are reports linking REM sleep behavior disorder with severe sleep bruxism, indicating a potential subclinical consequence of REM disturbances (118).

In patients with altered consciousness states such as coma, bruxism episodes can occur at varying levels of consciousness, and typically resolve with improvements of consciousness levels, coinciding with the return of the sleep–wake cycles (119). Studies on patients with acquired brain injury also exhibit a high incidence of bruxism, indicating its association with altered states of consciousness (120).

Overall, the sleep architecture of patients with sleep bruxism appears to be disrupted, with bruxism episodes potentially linked to disturbances in the sleep–wake cycle. However, the variability of these changes may depend on the severity of the condition and its interaction with other sleep disturbances.

### 6.3 Tooth clenching and limb muscle activity

Voluntary teeth clenching, also known as the Jendrassik maneuver, enhances tendon reflexes in the upper and lower extremities by facilitating peripheral monosynaptic reflexes (H-reflexes) (121). This facilitation is likely influenced by central motor control and sensory feedback from the muscle spindles and periodontal ligament (122), suggesting a possible role of orofacial proprioception in the regulation of limb muscle function. In humans, H-reflexes in muscles like soleus and the flexor carpi radialis are boosted during voluntary tooth clenching (122, 123). Conversely, tooth clenching may reduce deltoid muscle recruitment through presynaptic inhibition of muscle spindle inputs to the motor neurons of the deltoid muscles (124); while tooth grinding during sleep could inhibit the H-reflex of the gastrocnemius muscle (125).

The relationship between muscle reflexes and sleep–wake states remains unclear despite evidence suggesting a link to arousal states. During voluntary clenching, corticospinal pathways are implicated in increased recruitment of hand and leg muscles (121, 126). Additionally, teeth clenching neural circuitry seems to be more complex than the one involved in hand clenching (127). Muscle recruitment facilitation during teeth clenching may involve both cortical and subcortical areas for the upper extremities, while only subcortical areas are involved for the lower extremities (121). Motor-evoked response facilitation using transcranial magnetic stimulations have shown that an anterior-medially current direction facilitates the motor responses of the finger muscles at early stages of voluntary clenching task, possibly by influencing the motor cortical circuitry (128, 129). The motor cortex may regulate the hand muscles at the beginning of clenching, while the spinal cord may stabilize this activation at the late phase of clenching. Additionally, activation of both the hand motor area and greater hand muscle excitability during the execution phase of clenching (36). This may be explained as teeth clenching neural circuitry, possibly the primary motor cortex, cingulate motor area or supplementary motor area, may activate the hang region in the primary motor cortex (36, 130). The role of clenching in the control of limb muscles is suggested to be necessary for body stabilization, as in tasks such as weightlifting.

Sleep disorders involving motor activity, such as sleep bruxism and periodic limb movements during sleep, often co-occur and exhibit temporal concurrence with electroencephalogram arousals, indicating shared neurophysiological mechanisms (131–134). Patients with sleep bruxism frequently exhibit RMMA, accompanied by limb movements during sleep, indicating a response to cortical arousal (109, 135). These episodes, especially when accompanied by limb movements, suggest that sleep bruxism may be linked to cortical arousal and neural pathways interconnected with limb muscle facilitation.

## 7 Future insights and clinical implications

The understanding of the neural circuitry and neurotransmitters involved in bruxism holds the potential to develop future therapeutic strategies to effectively manage this condition.

While new technologies in both human and animal studies are shedding light on some aspects of the neural pathways involved in bruxism, many aspects remain unclear. This suggests the need for more studies to improve the treatment approach for this condition. Conducting more clinical studies that integrate neuronal activity, neuronal plasticity assessments, and electromyographic recording of the masticatory muscles would be valuable. Additionally, animal studies utilizing genetic tools and advanced techniques in neuronal tracing and manipulation can provide crucial insights.

Priority should be given to investigating the function of the trigeminal system under stress and anxiety conditions to better comprehend the development of masticatory muscle hyperactivity and bruxism. Exploring the roles and neuronal connectivity of areas such as Vmes, Peri5, Vpdm, and Vmotor could significantly advance our understanding in this field.

## 8 Conclusion

Bruxism is a multifactorial condition that can be present during wakefulness, sleep, or wake–sleep cycles, potentially involving distinct neural pathways for each subset. Higher brain regions responsible for processing stress and anxiety, along with nuclei within the trigeminal system, exhibit extensive neural connectivity that likely contributes to the pathophysiology of bruxism behaviors. Disruptions in serotonin and dopamine systems are implicated in the development of this disorder, highlighting their potential roles in its pathophysiology.
